# Development, Characterization and Sensory Evaluation of an Extruded Snack Using Fig Molasses By-Product and Corn Semolina

**DOI:** 10.3390/foods12051029

**Published:** 2023-02-28

**Authors:** Ismail Boluk, Seher Kumcuoglu, Sebnem Tavman

**Affiliations:** 1 Department of Food Engineering, Graduate School of Natural and Applied Sciences, Ege University, Izmir 35100, Türkiye; 2 Food Processing Department, Kösk Vocational School, Aydin Adnan Menderes University, Aydin 09100, Türkiye; 3 Faculty of Engineering, Department of Food Engineering, Ege University, Izmir 35100, Türkiye

**Keywords:** snack, extrusion, pasting, optimization, RSM, sun dried fig, molasses, by-product corn semolina

## Abstract

The effects of extrusion process parameters on the physicochemical, pasting and technological properties of ready-to-eat snacks were evaluated. The aim was to develop fortified extruded products with fig molasses by-product powder (FMP), which is created as a result of the production of fig molasses, is not currently used in food industry, and may cause environmental problems. The feed humidity was changed to 14–17–20%, the die temperature was 140–160–180 °C and the ratio of FMP was 0–7–14% at a fixed screw speed of 325 rpm. The study showed that adding FMP to extruded products had a significant effect on colour properties, water solubility and water absorption index properties. İncreasing the FMP ratio had a significant reducing effect on dough properties of non-extruded mixtures such as peak viscosity (PV), final viscosity (FV) and setback viscosity (SB). The optimum conditions for the production of snacks were found to be 7% FMP, 155.44 °C die temperature and 14.69% humidity. It was determined that the estimated values of water absorption index (WAI) and water solubility index (WSI) for the products manufactured under ideal extrusion conditions were close to the obtained values, and that there was no significant difference between the estimated values of the other response variables and their actual values.

## 1. Introduction

The food processing industry generates a huge amount of waste by-products. The use of industrial by-products enhances food process profitability and sustained food production [1]. Though the waste generated from food processing possesses a significant issue, the positive benefits of waste materials for human and animal species cannot be denied [2]. Internationally, “Zero Waste Alliances” are being formed to recycle nutrients and decrease environmental damage. There is a significant amount of ongoing research attempting to convert this food waste into valuable and nutrient-rich food components. Food waste is being utilized more and more as the ultimate substrate for the recovery of useful components and in the manufacture of innovative, high-quality products. For effective energy usage, nutritional recovery, and long-term food preservation, the food industry uses a variety of processing procedures for product generation, which include drying, boiling, and molasses production. Molasses is widely used as a preservative in storing perishable foods; it is produced by a variety of methods, including evaporation, condensation, and crystallization of fruit juices such as apple, mulberry, grape, fig, etc. [3]. Molasses contains the nutrients of the fruit from which it is obtained as a concentrate. For the production of fig molasses, generally defective fresh or dried fruits that cannot be packaged are used. The figs are first washed to remove impurities such as dirt and pesticides.

The dried figs of the washed figs are kept in water to absorb the water. The fruits are then disintegrated. Water-soluble substances are allowed to pass into the water by diffusion. Then the pulp and the must are separated from each other by filtration. The separated must is treated for a certain period of time with pectin-degrading enzymes to improve the yield, colour and taste. The clarified must is removed from the residue by centrifugation, filtration, or siphoning. Under a certain temperature and vacuum, it is concentrated to the desired Brix degree [4]. Fig fruit and fig molasses play a significant role as dietary supplements due to their high nutritional content. Carbohydrates, minerals, and organic acids are the major constituents of molasses [5]. Molasses also contains trace amounts of protein, amino acids, phenolic compounds, and flavonoids [5]. Phenolics and flavonoids have additional health benefits due to their antioxidant and antimutagenic properties [6]. There is a tremendous amount of waste generated during the processing of fig products and fig molasses. Fig pulp is the major waste generated, one that undergoes putrefaction with time and that can present serious concerns if not properly managed. Data from the literature illustrates the role of foods made with fig fruit in promoting health. However, there is no research on the topic of re-using fig by-products and molasses pulp in the food industry in the literature. Research is required to support the management and recycling of wastes generated during the production of fig molasses.

Fruits and vegetables are high in dietary fibre (DF), which supports human physiological activities by lowering cholesterol, reducing hyperlipidaemia and hypertension, and protecting gastrointestinal health [7]. Wastes from fruit and vegetable products are abundant and inexpensive sources of dietary fibre [8]. They are inexpensive, available in large quantities, and are characterized by high fibre content with high water-binding capacity, while being relatively low in enzymatically digestible organic matter [9]. By-products can be employed to change the physicochemical qualities of the human diet due to their high fibre content and contrasting dietary fibre features [10]. Expanded snacks and cereals with high dietary fibre content are receiving increased attention as a way to boost fibre intake. Traditionally, snacks are foods that are consumed right after being unpacked. The majority of customers’ daily nutritional and caloric demands can be satisfied by snacks [11]. Extrusion is a versatile, low-cost, and highly effective process, and it has no wastewater issue [12]. It enables the production of ready-to-eat food products by shaping, baking, and texturing by means of forcing them through a die. It is therefore ideal for incorporating food industry by-products into new snacks [13]. A twin screw extruder was used in this study to add fig molasses product waste (pulp) to corn semolina with a high starch content to produce snack foods with a high nutritional value and dietary fibre content. Additionally, the study sought to determine how the primary physicochemical features of extrudates were affected by extrusion variables such as die temperature, feed moisture, and fig molasses production waste content.

## 2. Materials and Methods

### 2.1. Material

Pre-washed sun dried figs that tested negative for aflatoxin under UV light were divided into 2 or 4 parts with the help of scissors to prevent harm to seeds like in a manner similar to the commercial production of fig molasses. Dried figs were soaked in 45 °C water for 5–6 h. The volume of water used was 2–3 times of the final fig weight. To make molasses, the juice was filtered, and the figs were again treated in water as described above. The process was repeated till the final water-soluble dry matter content of figs fell between 2–7%. The pulp obtained was layered as a thin film on a tray and dried in a rotary forced-air tray dryer (Eksis Industrial Drying Systems, Isparta, Türkiye) at 45 °C to a moisture content of 5–9%. Samples obtained on different days were mixed before grinding. The oil of the seeds was reduced from 30.70 ± 2.51 to 19.65 ± 0.15% by a mechanical pressing method with a screw press (Karaerler Machinery Energy, Ankara, Türkiye). The protein content was 13.50 ± 0.1%, the moisture content 5.19 ± 0.24%, the ash content 4.90 ± 0.27%, and the total dietary fiber content was determined to be 53.01 ± 0.51% listed in Table 1. It was processed in a lab-scale hammer mill (FT2, Armfield Technical Education, Ringwood, UK) to a fine powder that could pass through a 0.5 mm (35 mesh) sieve. For future testing and analysis, it was kept airtight at −18 °C in polyamide bags (PA) to avoid oxidation and other degradation. Corn grits were obtained from Semolina Corn Semolina Food Industry and Trade Inc. (Samsun, Türkiye). It contained 5.0–9.0 g protein, 70.0–80.0 g carbohydrate, <1 g fat, <0.7 g dietary fiber, <14 g moisture, and 0.2–0.6 g ash per 100 g. Particle distribution of grit was >500 µm for 50–65 g, and >300 µm for 35–50 g per 100 g of grit.

### 2.2. Preparation of the Mixture Formulation

Corn semolina and fig molasses wastes in dry powder form were mixed at 100:100, 93:7 and 86:14 ratios. The moisture contents were adjusted to 14%, 17%, and 20% by spraying the calculated amount of distilled water. The mixture was then placed in sealable PA bags and kept in the refrigerator at +4 °C overnight to equilibrate the humidity. A halogen moisture analyser was used to determine the moisture content of the mixture before extrusion (MOC63, Shimadzu, Kyoto, Japan).

### 2.3. Extrusion

**Figure 1:**
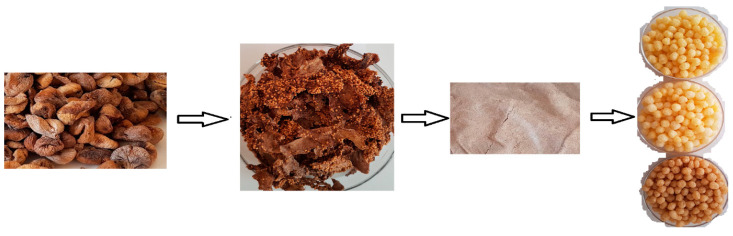
Sun dried fig fruit, dried by-products of molasses, and in ground form (FMP), as well as extruded samples of corn semolina-FMP, respectively.

A laboratory scale co-rotating twin screw extruder with a die diameter of 2.5 mm and a barrel-length-to-screw diameter ratio of L/D 25:1 (Feza Gıda Müh. Makine Nakliyat ve Demir Tic. Ltd. Şti., İstanbul, Türkiye) was employed in the study. The screw speed and feed rate were set to 325 rpm and 255 g/min. The barrel temperatures for the first three zones were set at 60 °C, 100 °C, and 130 °C. The production steps are shown in Figure 1. Ten minutes after the system’s operating parameters stabilized, the extruded products began to be collected and were dried in conventional oven at 45 °C until the final moisture content was 5–8%. The final products were placed and kept at ambient temperature until analysis.

### 2.4. Analysis of Flour Samples

Ash determination was carried out according to Method AOAC 920.153. The protein content of fig molasses pulp powder samples were measured according to AACC method no 46-12 (AACC, 2010). Moisture determination was determined according to AOAC 925.10, and fat content was determined with reference to AOAC 948.22.

### 2.5. Analysis of Total Dietary Fiber

The total dietary fiber contents of raw materials and extruded products were determined according to the AACC, 32-07 enzymatic gravimetric total dietary fiber assay procedure using a total dietary fiber kit (1.12979, Merck, Darmstadt, Germany)

### 2.6. Analyzing the Attributes of a Product

#### 2.6.1. Diametric Expansion Ratio

The cross-sectional expansion ratio (ER) was computed by dividing the exudates’ diameter by the die’s diameter, which was 2.5 mm. The extrudate diameter was calculated by averaging the diameter length of 10 randomly chosen samples. ER = extrudate diameter (De)/die diameter (Dd)

#### 2.6.2. Bulk Density (g/cm^3^)

Bulk Density (BD) was determined according to Equation (1):

BD (g/cm^3^) = 4M/πD^2^L (1)

D: Extrudate diameter (cm);

M: Weight of extrudate at length L (g);

L: Length of gram extrudate (cm) measured using a digital caliper (Mitutoyo Co., Kanagawa, Japan).

All the experiments were done in triplicate.

#### 2.6.3. Water Absorption Index (WAI) and Water Solubility Index (WSI, %)

WAI was calculated according to the procedure outlined by Anderson, Conway, and Peplinski (1970) [14]. The experiment was done in triplicate. WAI was calculated according to Equation (2):

WAI (g/g) = weight gain of gel/dry weight of sample(2)

The weight of the solid particles in the supernatant produced from the WAI test was divided by the weight of the original sample to determine the WSI, as calculated with Equation (3):

WSI (%) = [M_1_/M_2_] × 100 (3)

M_1_: Weight of solids dissolved in the supernatant (g);

M_2_: Dry weight of the extrudate (g).

#### 2.6.4. Colour Parameters

A Minolta spectrophotometer was used to measure the colours of the extrudates (CM-700d, Konica Minolta Sensing, Tokyo, Japan). For each sample, an average of 3 measurements were taken. Results are expressed using the CIE L, a, b colour space.

#### 2.6.5. Hardness

Texture Analyzer TA-XT plus, (Stable Micro Systems, Haslemere, UK) and a 5-blade Kramer shear cell were used to measure hardness. The samples’ sizes were modified to 3 cm in length to ensure suitability in measurements. A total of 3 replicates were performed for each sample with a 50 kg load cell, and the average results were calculated. The maximum cutting force applied was considered hardness. Measurements were performed at a test speed of 2 mm/s, a post-test speed of 10 mm/s, and a test height of 5 mm.

#### 2.6.6. Microstructural Characteristics

Snacks were cut into 3.0 mm thick pieces and samples were mounted on carbon discs using a silver tape and covered with a gold layer in a vacuum sublimator (Leica EM ACE600, Wetzlar, Germany). SEM (Thermo Scientific Apreo S, Waltham, MA, USA) was used to examine the cross-section of samples at different magnifications (48× and 500×) at 20 kV acceleration voltage.

#### 2.6.7. Pasting Properties

Pasting properties (pasting temperature and viscosity) of extruded samples were measured by using a controlled-stress rheometer (TA DHR3, TA Instruments Inc., New Castle, DE, USA). The pasting temperature (PT), peak viscosity (PV), final viscosity (FV), breakdown viscosity (BV), and setback viscosity (SV) of the samples were recorded. Evaluations were obtained in triplicate.

#### 2.6.8. Sensory Characteristics

All participants were informed about the content (ingredients) of the samples and their consent was obtained prior to their participation in the sensory analysis. The sensory evaluation of samples was conducted by 7 trained panellists who consumed the expanded snacks. All of the panellists were recruited from among graduate students between the ages of 24 and 30 years old (4 women and 3 men). Samples were assessed based on a nine-point hedonic scale in which “dislike extremely” was scored as 1 and “like extremely” as 9. At the beginning of the overall acceptability assessment, clear definitions of terms related to the texture and taste characteristics of the products, such as corn, fig, rancid, and burnt tastes were given to the panellists, and care was taken by the panellists to understand the meanings of each feature. Hardness was measured by the amount of force required to break a sample on the first chew with molars, with reference to Uno Sandwich Bread, which was defined as being of density level 1. Crispness was measured by the intensity of audible noise at first chewing with molars; Kellogg’s breakfast cereal’s degree of crispness was 7.5. Corn flavour was measured by the grain flavour associated with corn by the reference to Star Krak Plain Corn Snack, the density of which was 8. Fig flavour, the flavour associated with fig fruit, was 9 for sun-dried figs. Rancidity, characterized by spoiled fat and oil, was 2.5 for sunflower oil kept in the microwave for 3 min at high degree. Burnt was associated with overcooked grains by reference to Toasted French Bread Tips, which had a density of 8.

#### 2.6.9. Experimental Design and Statistical Analysis

The experimental data were arranged using the Box Behnken design. RSM was carried out using the commercial statistical application Design-Expert version 7.0.0 (Statease, Inc. Minneapolis, MN, USA). The experimental design and the independent variable codes, each with three levels, are shown in Table 2 and Table 3. For the dependent variables, quadratic polynomial regression models were created using the statistical program Design-Expert v 7 to fit each response to the experimental data. The experimental response was represented by the predicted response functions (Y), while A, B, and C were independent variables determined by a quadratic polynomial equation.

Y = b_0_ + b_1_A + b_2_B + b_3_C + b_11_A^2^ + b_22_B^2^ + b_33_C^2^ + b_12_AB + b_13_AC + b_23_BC

The polynomial model’s coefficients are represented as b_0_ (interception), b_1_, b_2_, and b_3_ (linear effects), b_11_, b_22_, and b_33_ (quadratic effects), and b_12_, b_13_, and b_23_ (interaction effects). To ascertain the impact of each factor on the responses and the model’s fit, analysis of variance (ANOVA) was used. To evaluate the ideal conditions and show the relationship between each factor’s reaction and its experimental levels, appropriate polynomial equations were written as surface plots. For statistical analysis and extrusion process optimization, Design-Expert version 7.0.0 (Statease, Inc., Minneapolis, MN, USA) and IBM SPSS Statistics 22 were employed.

## 3. Results and Discussion

The statistical significance of the response variables was investigated, and the best model selected, using regression analysis and ANOVA (*p* < 0.05). Using coefficients of determination (R^2^) and coefficient of variation, the model’s fit was evaluated (CV). For all response variables, the lack of fit was not statistically significant, showing that these models are reliable predictors of responses (Table 4). R^2^ measures how well the empirical model fits the actual data [15]. Excluding WSI and WAI, R^2^ values above 0.75 were achieved, indicating that the model can be applied to the data. For all response variables, the coefficient of variation (CV), which describes the distribution of data, was less than 14%.

### 3.1. Diametric Expansion Ratio (ER)

The quadratic model for ER’s analysis of variance (ANOVA) is presented in Table 4. With a variance coefficient of 2.87%, the coefficient of determination (R^2^) and adjusted R^2^ values are 0.985 and 0.971, respectively. F value was significant for ER at 67.85 (*p* < 0.005), but lack of fit at 0.177 (*p* > 0.05) was not. According to the regression analysis in the table, the regression model obtained for ER was significant (*p* < 0.01). In terms of the coded levels of the variables, the quadratic model for ER that resulted from the regression analysis was created as follows, as Equation (4):

ER = 3.92 − 0.34A − 0.70B − 0.33C + 0.17AB + 0.23AC − 0.17A^2^ + 0.27B^2^ − 0.22C^2^ (4)

where; A: Temperature (°C). B: Fig molasses by-product powder level (%). C: Moisture content (%wb).

**Figure 2:**
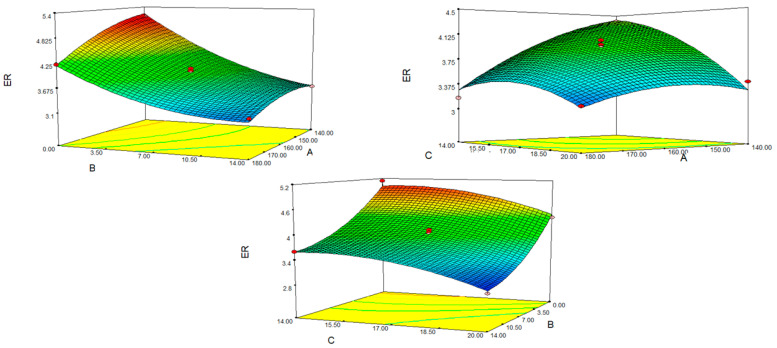
Response surface plots for the effect of fig molasses pomace content, moisture and temperature on the expansion ratio (ER): (A) temperature (°C); (B) fig molasses by-product powder level (%); (C) moisture content (%wb).

The expansion ratio reveals how much spattering is seen as it passes through the die [16]. The material expansion is attributed to the bubble expansion caused by the pressure differences between melt exiting the extruder, atmospheric pressure, and die swelling [17]. The study found that both linear (*p* < 0.001) and quadratic effects (*p* < 0.05) were significant. The intensity of the starch gelatinization is an important factor affecting the expansion ratio [18]. Some discrete factors control the end product’s rate of expansion, including the limited access of water to starch due to the dietary fibre and the prevention of air-filled bubbles from bursting cell walls before they are inflated by FMP fibres [19]. For these reasons, increasing the amount of fig pulp decreased the expansion rate of the final product. A similar observation was seen with passion fruit peel when mixed with rice flour and tomato puree, along with corn semolina, flaxseed, and corn flour [20,21,22]. In extruded products produced by mixing barley flour and carrot pulp, linear and quadratic effects of pulp amount were found to be significant, and it was reported that ER decreased as the amount of pulp increased [23]. Navarro Cortez et al. (2016) incorporated orange pomace in the development of extruded snacks; the orange pomace negatively affected the expansion index due to the increased amount of fibres, which ends up reducing the amount of starch and compromising the gelatinization of the mixture [24]. İn extruded snacks based on rice flour mixed with cashew apple pomace powder at levels ranging from 0 to 25%, fruit content negatively affected expansion ratio [25]. The quadratic term of temperature played an important role, since the increase in temperature from 140 °C to 160 °C, which is in the manufacturing range, resulted in an increase in expansion ratio followed by a decrease in its value (Figure 2). This could be because higher barrel temperatures increase the degree of gelatinization as well as the amount of superheated steam, causing the snack to expand more [26]. Increased barrel temperatures reduce melt viscosity, which promotes bubble growth during extrusion [27]. The expansion ratio decreased at temperatures higher than 160 °C due to degradation of the starch structure. Similar results were observed by other researchers when corn semolina and food manufacturing by-products were mixed, which showed that ER rose when temperature was increased from 146 °C to 180 °C but dropped between 180 °C and 210 °C [26]. In another study, when maize bran was added to corn flour, a similar outcome was seen [28]. Similarly, where barley flour and carrot pulp were combined, ER increased as the temperature rose from 120 °C to 145 °C but declined after that point due to the breakdown of starch [23]. The viscosity of the dough mass was found to reduce during high-temperature procedures, increasing the linear velocity in the die. There is an inverse relationship between dough consistency and expansion [29]. However, depending on the type and moisture content of starch, the decreased expansion ratio over the threshold temperature may be caused by increased starch melting, softening, and structural damage that cannot survive excessive evaporation [28]. This observation coincides with the work done by Thakur et al. 2017, who also noted a negative correlation between extrusion temperature and expansion rate [30]. With increasing extrusion temperature, the rate of expansion of extruded maize grain reduced significantly. This shows that, depending on the composition and rheological characteristics of the input materials, the influence of greater extrusion temperature might be either positive or negative upon the expansion characteristic [12]. One of the primary elements affecting expansion is feed humidity [27]. In the present study, the regression coefficients revealed that the moisture content has a quadratic and linearly negative effect (*p* < 0.05). In general, the expansion ratio fell as humidity increased, although occasionally, there was a small increase followed by a decrease. Similar observations were noted in the investigations of Singha et al. (2018) and Shirazi et al. (2020) [11,23]. The lower the moisture content, the greater the friction, and the creep force, resulting in a higher pressure in the die, leading to greater product expansion due to a greater pressure difference at the die exit [31]. Conversely, as humidity increased, the elasticity and temperature of the dough decreased due to decreased friction between the dough, screw, and barrel, which negatively affected gelatinization and reduced expansion [32]. Increasing the moisture content during extrusion may reduce the elasticity of the dough through plasticization of the melt, resulting in decreased gelatinization, expansion, and density [33]. Similarly, it was shown in a study that looked at a Jerusalem artichoke–rice flour–corn flour that ER initially increased (13–18%) with an increase in feed humidity but reduced (18–24%) above this rate [34]. In the extruded product investigation with carrot pulp-rice and legume flours, the ER increased as the moisture content increased, and then the elasticity of the dough dropped and the ER fell as the humidity increased [35]. It has been found that the ER values of extruded products, in which food processing wastes such as lean soy flour, germinated brown rice flour and mango peel fibres are mixed with corn semolina, first increase with the increase in humidity, but decrease if it is above 15% [26].

### 3.2. Bulk Density

An essential characteristic of extrudates is bulk density. The expanding attribute that happens when extrudates exit the die section has a common effect on BD [36]. In the present study, Bulk density was significantly influenced by barrel temperature and starting moisture content (*p* < 0.001). Table 4 provides the results of the quadratic model’s analysis of variance (ANOVA). The coefficient of determination (R^2^) and adjusted R² values obtained with 9.0% coefficient of variance are 0.932 and 0.891, respectively. For BD, an F value of 22.88 was significant (*p* < 0.005), while a lack of fit of 0.300 was not significant (*p* > 0.05). The generated regression model was significant for bulk density, according to the regression analysis in Table 4 (*p* < 0.01). The quadratic model for bulk density (BD) based on the coded levels of the variables is presented in Equation (5):

BD = 0.090 − 0.020A + 0.012B + 0.022C − 0.022AC + 0.011A^2^ + 0.013C^2^ (5)

**Figure 3:**
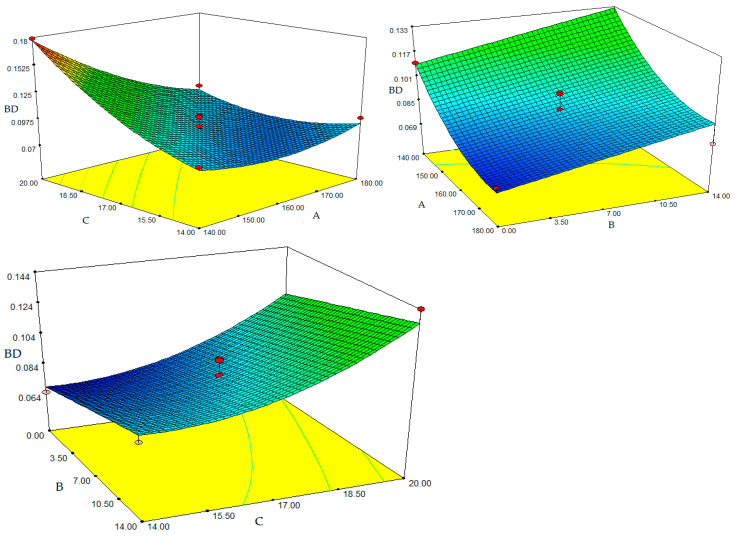
Response surface plot for the effect of fig molasses pomace content, moisture and temperature on the bulk density (BD): (A) Temperature (°C); (B) Fig molasses by-product powder level (%); (C) Moisture content (%wb).

The negative linear effect of die temperature and the positive linear effect of FMP content and moisture content on BD were significant (*p* < 0.005). The BD was influenced by the quadratic temperature and moisture content terms (*p* < 0.05). Temperature and moisture content interactions were also found to be significant (*p* < 0.005). Since the majority of extruded items are filled by weight rather than volume, the BD is a crucial quality attribute from a commercial perspective. If the BD fluctuates while the pack is being made, it is either underfilled or overfilled. A thorough control of BD will guarantee that the product’s texture is within the necessary quality parameters and will also verify the packaging [11]. The BD value of the snack was between 0.064 and 0.179 g/cm^3^. The effect of the quadratic term of the die temperature was also found to be significant (*p* < 0.05). Although the bulk density value grew along with FMP content and moisture content, it declined as the die temperature increased. This is because running the extruder at a higher temperature causes the water in the extruder sleeve to become superheated, which promotes bubble formation and lessens melt viscosity. The FMP-corn grits snacks’ expansion ratio increases and their bulk density decreases as a result [37,38]. Figure 3 displays three-dimensional graphs showing the relationship between feed moisture and bulk density. The presence of moisture has a secondary impact on BD. As the feed humidity rose, it was found that the bulk density rose as well. This is because a decrease in feed humidity causes the melt’s viscosity to decrease, which in turn causes the extrudate’s bubbles to collapse after expanding at the die exit, increasing the bulk density [12]. BD may continue to rise as a result of the dough’s decreased elasticity and its smaller expansion [39]. In the published literature, similar circumstances were seen in [11,37,40]. According to Ding et al. (2015), moisture content has a major impact on how much extruded items expand [27]. The bulk density was shown to be significantly affected by the amount of FMP (*p* < 0.005). The bulk density grew along with the amount of FMP. This is so because fruit peel flours have a high fibre content that may prevent extrudates from expanding radially [21]. This might also be because non-starch polysaccharides can bind more water than can starch and protein [41]. Additionally, it was observed that in mixes of both maize flour with pineapple pulp and carrot pulp with corn starch, the extrudates’ densities increased [42].

### 3.3. Colour Parameters

**Figure 4:**
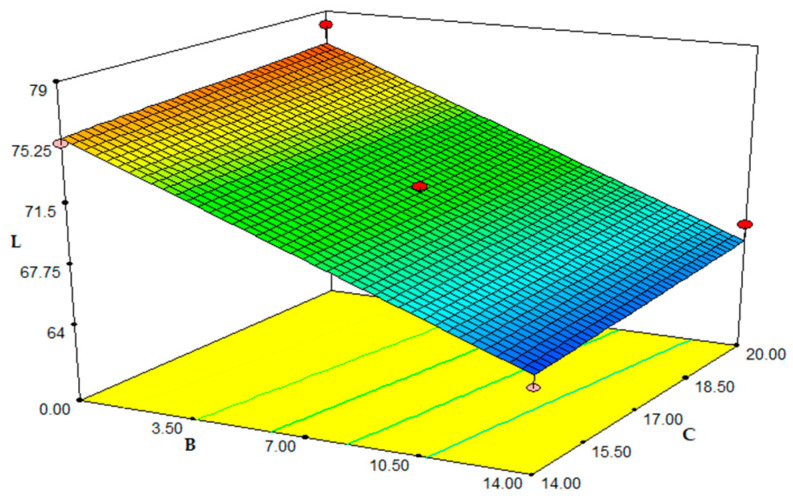
Response surface plot for the effect of fig molasses pomace content, moisture and temperature on the colour: (A) temperature (°C); (B) fig molasses by-product powder level (%). L: Lightness.

Colour is a crucial physical characteristic that should be documented for extruded items and a crucial quality component that is directly related to the acceptability of food products [12]. L* values were used to create multiple linear regression equations. Figure 4, below, provides a visual representation of the value changes. Table 4 provides the linear model of L’s analysis of variance (ANOVA). The *p*-value for the lack of fit was 0.266, which was insignificant. The model of L*, the colour parameter of the extrudates, was linear (*p* < 0.001) and significant. The experimental results are in good agreement with the estimated values, as shown by the observed R^2^ and adj R^2^ values of 0.942 and 0.929, respectively. For the predicted model of the L* value, Equation (6) in the form of coded values can be created follows:

L* = 70.95 + 0.066A − 5.26B + 0.59C(6)

In the studied range, pulp content had a negative linearly significant effect on L* value (*p* < 0.001). Temperature and humidity had no observable influence (*p* > 0.05). The samples’ lightness levels (L*) were measured in the range of 64.31 and 78.01. In the range of values being tested, L* value dropped as FMP content in the combination grew (Figure 4). Reducing sugars and proteins (amino acids) may combine at high processing temperatures to produce non-enzymatic browning due to Maillard reactions [43]. Corn grits have a large amount of reducing sugar, however [44], and the pulp also has a large amount of protein. Similar outcomes were found in tests with dry ethanol production wastes [43], rice flour and rice husk, apple pulp, and corn semolina [11]. It was observed that the L* value increased as the moisture content increased, which is similar to our investigation’s findings [45].

### 3.4. Hardness

The force needed to break the extrudates of corn semolina and fig molasses pulp was measured to assess their textural characteristics. The harder the sample, the greater the force needed to break it. The goods’ hardness levels ranged from 11.51 to 37.16 kg. These values were found to be comparable to the hardness outcomes of extruded goods obtained with rice flour and mushroom powder [37]. However, it was discovered to be greater than the values in the study using maize flour, wheat flour, and mushroom powder [46]. Again, the literature contains many results that are higher than these values. Measurements of breaking strength vary greatly in the literature due to varying compositions, probes utilized, and methodologies [37]. In the present study, Table 4 (ANOVA) shows that the stiffness regression model’s *p*-value is <0.0001 significant, the lack of fit’s *p*-value is 0.148 (*p* > 0.05), and the R^2^ and adj R^2^ values are, respectively, 0.903 and 0.871, which shows a good fit. Equation (7) for the predicted model of hardness in the form of coded values may be designed as follows:

Hardness = 19.16 − 3.2A + 5.00B + 8.27C + 5.60C^2^ (7)

**Figure 5:**
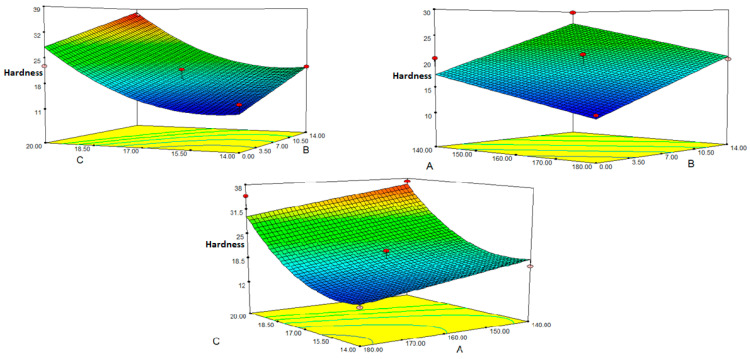
Response surface plot for the effect of fig molasses pomace content, moisture and temperature on hardness: (A) Temperature (°C); (B) Fig molasses by-product powder level (%); (C) Moisture content (%wb).

According to ANOVA, hardness was significantly influenced by barrel temperature, FMP content, and moisture content (*p* < 0.05). It was shown that the working range’s hardness rating was positively correlated with the amount of FMP present. This outcome is consistent with a rise in both FMP quantity and bulk density. Due to the strong association between product density and hardness, high-density products naturally produced high hardness. Because the pulp absorbs excess moisture, an increase in FMP quantity could result in a hardening of the material [32]. Additionally, the non-starch polysaccharides in the FMP damage the enlarged snacks’ overall cell structure, which has an adverse effect on the growth of gas cells. With tomato puree and barley flour, similar outcomes were seen [12]. In extruded products made from a blend of sweet potato flour, rice flour, and corn flour, the hardness increased as the sweet potato content increased and dropped as the die temperature rose [34]. Extruded morning cereals made of whole-grain wheat flour and jaboticaba husk were found to have a similar circumstance [47]. As the temperature rises, the melt’s viscosity reduces, allowing bubbles to form in the melt and lowering the hardness value as a result [48]. Once more, this circumstance was related to bulk density. The bulk density reduced as the temperature rose, and the hardness also decreased. Similar circumstances were noted in the investigation using barley flour and carrot pulp [23]. Similar to this finding, it has been found that, as the temperature rose, the hardness of snacks made from brown rice semolina decreased [49,50]. The opposite pattern, however, was seen in extruded items made from barley and tomato puree mixed with rice flour, pineapple pulp, and lentil flour [12,40]. The effect of the square term for the moisture content was significant, as evidenced by the F and *p* values for the square term of the moisture content being 15.42 and 0.002 (*p* < 0.005), respectively. The hardness value of the operating range increased with the moisture content (Figure 5). Because moisture inhibits bubble development, the structure is denser and the texture is tougher [51]. As a result, there will be less friction and shear between the food material and the extruder screw. More water in the materials will act as a lubricant in the screw. With an increase in moisture levels, the hardness of extruded items made with rice flour and mushroom powder also rose [37]. Similar circumstances were noted in the investigation using barley flour and carrot pulp [23]. As well, similar results were seen in the study that looked at how gelatinized rice flour affected the physicochemical characteristics of ready-to-eat expanded snacks [52]. In the investigation of corn flour-wheat bran extruded goods, a similar circumstance was seen [48]. However, research in the literature indicates that the hardness declines as the moisture content rises [35].

### 3.5. Pasting Properties

The pasting properties of food describe the changes that occur in the food when heat is applied to the food in the presence of water. These changes affect the texture, digestibility, and end-use of the food product. The pasting temperature (PT) is defined as the lowest temperature to initiate swelling and gelatinization of starch. Pasting viscosity refers to the degree of starch gelatinization [53].

The final viscosity defines the viscosity measurement at the end of the test, and break-down viscosity signifies the ability of the sample to resist thermal and mechanical stress. The means of pasting characteristics, pasting temperature (PT), peak viscosity (PV), breakdown viscosity (BV), and setback viscosity (SB) of corn semolina and its enhanced blends with fig molasses by-product powder (FMP) are provided in Table 5. The materials’ PT, which represents the lowest temperature needed for gelatinization, varied between 70.62 and 74.1 °C. In the study in which corn kernels obtained in different sizes were examined, PT varied between 75.1 and 79.0 °C [54]. The pasting temperatures of corn grit-buckwheat blends ranged from 77.5 °C (for 0% buckwheat flour level) to 86.1 °C (for 10% buckwheat flour level) [55]. The increasing concentration of FMP led to a significant (*p* < 0.05) increase in PT. Pasting temperature would rise due to delayed or limited swelling and amylose leaching [56]. A steady increase in pasting temperature was observed with increasing mung bean flour content, which could be attributed to delayed peak time or swelling of granules [57]. Higher gelatinization temperatures (as determined by higher pasting temperatures) have been reported for corn grit, flours, starch with higher protein content, and rice flours in the presence of greater amounts of pulse proteins [58]. Peak time, in which the viscosity of the material reaches a maximum value, is significantly different for FMP-added blends. Corn grits that are blended with FMP reach maximum viscosity over a long duration; the addition of FMP has an accelerating effect (*p* < 0.05), which would be caused by the lower starch content of the formulations. The behaviour of viscosity developed during the cooking cycle reflects the capacity of modified corn grit to absorb water and swell as the slurry is heated [59]. The increase in viscosity with increased temperature may be attributed to the removal of water from the exuded amylose by the granules as they swell. The highest PV was observed in plain corn grits, in which 14% of blends displayed the lowest PV, whereas the PV of the samples changed between 351.4–1195.56 cP. FMP has a decreasing effect (*p* < 0.05). Similar trends were seen in the FV (a material’s ability to form a viscous paste) and SB (a measure of retrogradation tendency or syneresis of flours upon cooling of cooked flour pastes) [57]. Wheat flour added to grape extract powder also showed diminishing peak viscosity [60]. Fibre may compete with starch for free water, thus decreasing the occurrence of gelatinization and PV. However, there was also an overall loss in viscosity with the enhancement of buckwheat flour amounts in the corn grit blends, which might be due to the lesser degree of starch gelatinization [55]. It is possible that an increase in non-starch compounds is the reason for the poorer resistance of corn grit-FMP mixes to heating and shear mixing that, in turn, leads to reduced viscosity [61]. FMP has a reducing influence on PV, FV, and SB; the effect on PV and FV would be impacted by an increase in non-starch content in the formulations, which would result in less water being available for starch during the swelling phase. Nevertheless, falling SB values indicate the formation of a more stable structure with increasing FMP.

### 3.6. Water Absorption Index (WAI)

Starch’s ability to absorb water is measured by the water absorption index (WAI), which can be used as a gelatinization indicator [62]. The dispersion of starch in excess water, which results from gelatinization and breakdown by extrusion, which separately reduces the molecular weight of the molecules of amylose and amylopectin, has traditionally been linked to water absorption [63]. Mixtures of FMP and corn semolina were found to have WAI values ranging from 3 to 5.012 g/g. According to Table 3 (ANOVA), the F-value for the general regression equation for the water absorption index was 7.67, the *p*-value was 0.0034 (*p* < 0.05), and the lack of fit value was 0.855 (*p* > 0.05), which was not significant. For WAI, the adjusted R^2^ was 0.55 and the coefficient of determination R^2^ was 0.64. Pulp and moisture content had a significant impact on WAI, although the temperature had little impact (*p* < 0.05). For the expected model of WAI, Equation (8) in the form of coded values can be created as follows:

WAI = 4.26 − 0.045A − 0.54B + 0.37C(8)

As the amount of pulp in the working range increased, the WAI of the extruded products decreased linearly (*p* < 0.05). The degree of starch gelatinization in the barrel may be impacted by the relative reduction in starch content caused by the addition of FMP, which may be related to a competition between pulp and starch for water absorption. Similar results were seen in the investigation using durum wheat flour and hazelnut flour that had been partially defatted [41]. When pea semolina was introduced to rice semolina in the trial, the WAI dropped with the addition of more peas, and it was claimed that this was because the starch ratio of the pea combination had been reduced [64]. According to the results of the investigation using barley flour and tomato pulp, WAI dramatically decreased as the proportion of tomato pulp rose [12]. In the examined range, the influence of the mixture’s moisture content was positively linear, and the WAI rose as the humidity level increased. In another investigation, the pulp obtained after the manufacturing of mandarin juice was extruded with rice flour, and a similar condition was seen [32]. According to the research using corn semolina and apple juice pulp, the WAI value improved as the moisture level rose [11].

### 3.7. Water Solubility Index (WSI)

The physical and chemical structure of the extruder feed components, which are comprised of protein, starch, and fibre, is altered as a result of the extrusion process. It is possible to detect the soluble component released from starch following extrusion; it is known as WSI and is frequently employed as a marker of molecular component breakdown [39]. An in vitro sign of excellent starch digestion is a high WSI. The degree of dextrinization and gelatinization is based on WSI [65]. It may also determine the level of starch conversion, which is the quantity of soluble polysaccharides released from the starch granule during extrusion [39]. The WSI values for FMP corn semolina extrudates ranged from 24 to 69%. The p-value for the general regression equation was found to be 0.0007 (ANOVA) significant (*p* < 0.005) for the water solubility index, while the lack of fit values was determined to be insignificant when 0.277 (*p* > 0.05). The regression analysis for WSI gave a linear model, with R^2^ and adj R^2^ values of 0.718 and 0.653, respectively. The lowest WSI value was achieved at 140 °C, 7% FMP, and 20% moisture content, while the highest values were obtained at 180 °C, 7% FMS, and 14% moisture. The following, Equation (9) in the form of coded values, may be developed for the projected model of the WSI:

WSI = 41.54 + 7.74A + 1.09B − 7.50C(9)

In the tested range values, regression analyses revealed that moisture content had a negative linear effect while die temperature had a positive linear effect (*p* < 0.05). This is because starch experiences less shear and fragmentation during extrusion at high moisture concentrations, resulting in a lower WSI. Similar circumstances have been seen in extruded snacks including pineapple pulp [1] and dried distilled alcohol manufacturing grains (corn semolina) [11]. On the contrary, several studies have shown that when moisture content increases, so does the WSI value. This is due to the requirement for a specific quantity of water for starch gelatinization [45]. As the gelatinization degree of starch increases with the increasing temperature, so does the amount of soluble starch, causing the WSI value to rise with increases in temperature [39]. Similarly, in the extrusion of cornmeal and soy protein isolate, the WSI value rose with increasing temperature [45]. Another research effort, using a combination of carrot, rice flour, and legume powders, found that the WSI value (110–130 °C) declined and subsequently climbed with increasing temperature [35]. However, it has been found in tests on barley and oat products that WSI diminishes with rising temperature [66].

### 3.8. Total Dietary Fibre Content

The results show the effect of FMP addition on the total dietary fibre content of snacks. The mean dietary fibre content was found to be 1.23 in the samples with no FMP content, 2.04% in the samples with 7% FMP content, and 4.82% in the samples with 14% FMP content. Changing FMP for corn grits resulted in a statistically significant increase in the total fibre content of the samples (*p* < 0.05). It has been shown that the addition of FMP can increase the total dietary fibre content of products by a multiple of 3.9. This was due to the high level of dietary fibre content of FMP. According to the findings, the generated extrudates with 14% added by-products are eligible for the nutrition claim “source of fiber” under Regulations (EU) No 1924/2006 and (EC) No 1047/2012 [67]. Snacks with additional FMP can be recognized as functional foods since increased fibre levels are linked to improved health. An approximately fourfold increase was observed in the dietary fibre content of extruded products in which beer waste was added to corn flour [27]. It has been observed that there is an increase in dietary fibre content in the products obtained by adding apple pomace, brewer’s spent grain and sugar beet pulp waste to corn semolina [68]. Stojceska et al., (2008) reported that incorporation of cauliflower trimmings into ready-to-eat expanded products significantly increased the dietary fibre [10].

### 3.9. Microstructural Characteristics

**Figure 6:**
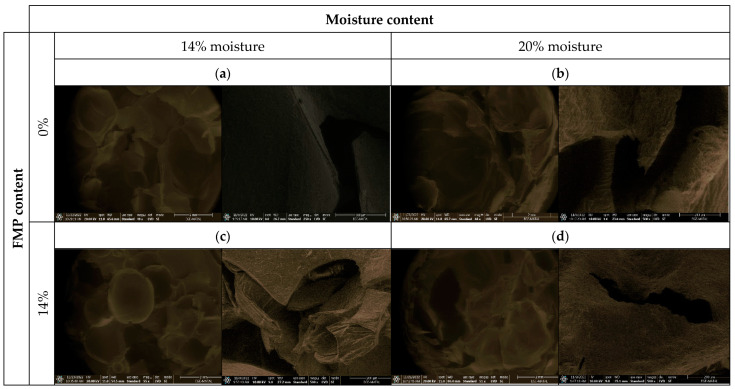
Effect of different combinations of feed moisture and FMP content on the microstructure of extruded snacks at 160 °C die temperature. Magnifications of 48–55× (**left**) and 250–500× (**right**) were made for each product. (**a**): 14% moisture 0%FMP; (**b**): 20% moisture, 0%FMP; (**c**): 14% moisture 14%FMP; (**d**): 20% moisture, 14%FMP.

Microstructural examination provides information about cell size, cell wall thickness, and spacing between cells in a food system, corresponding to the expansion and density indices [53] The internal structures of expanded extruded snacks observed via SEM are shown in Figure 6. In addition, the inner cell structures were evaluated under different magnifications to best characterize the structures. It can be verified that the existence of different processing conditions has a major influence on the inner cell structures of extruded snacks with varying concentration of FMP in similar conditions (at 160 °C die temperature).

The addition of fig molasses pulp affected cell formation in the extrudates; high percentages of pulp (14%) contributed to the formation of dense structures, with reductions in the average cell size, and with many holes in their walls. Trials without fig molasses showed a sample with few cells (but large ones), having relatively smooth surfaces and no holes between adjacent cells. which may be attributed to the presence of dietary fibre in FMP flour that acted as a nucleating agent and increased the number of air cells. In accordance with bulk density, expansion ratio and hardness data, extrudates with high levels of FMP have more bulk density, less expansion ratio and harder structures. In the study conducted with wheat husk-maize, it is stated that the degree of expansion depends on the amount of wheat bran in the formulation and is related to the cell size [48]. Similarly, adding more degreased cannabis powder or whole cannabis powder resulted in more and smaller cell structures [69]. In general, increased raw material moisture contents resulted in snacks with a dense texture and poor cell formation. This can be explained by the lower level of starch gelatinization, which contributes to the formation of the internal structure specific to extruded products. High moisture contents reduce the friction (mechanical shear) between the raw material and the extruder’s inner surface, lowering its temperature and consequently having a negative effect on gelatinisation [19]. Increasing feed moisture facilitates starch gelatinization, which leads to a thinner cell wall and larger air cell size, so the starch layer that contains the air cells becomes thinner and causes a decrease in hardness [70]. Similar results have been found for partially defatted almond powder and corn flour extrudates [70].

### 3.10. Sensory Characteristics

Sensory assessment is one of the most important parameters for food acceptance. The hedonic scale is used to determine the many aspects of sensory assessment. The sensory characteristics of extruded snacks were evaluated using a nine-point hedonism scale. The average score values of the goods derived from FMP ranged from 5.81 to 6.63, indicating that they were satisfactory. The findings revealed a highly significant (*p* < 0.05) model with a 1.55% coefficient of variation (CV) and a coefficient of determination (R^2^) of 0.927, with an adjusted R^2^ value of 0.857 (ANOVA). The model’s F-value of 14.87 and p-value of 0.0003 were both significant (*p* < 0.001) for overall acceptability, whereas the lack of fit value of 0.706 was not (*p* > 0.05). Equation (10) is built based on the coded values for the projected model of overall acceptability:

Overall acceptability: 6.40 − 0.070A − 0.14B − 0.045C + 0.17BC − 0.25 A^2^ + 0.19B^2^ − 0.24C^2^ (10)

**Figure 7:**
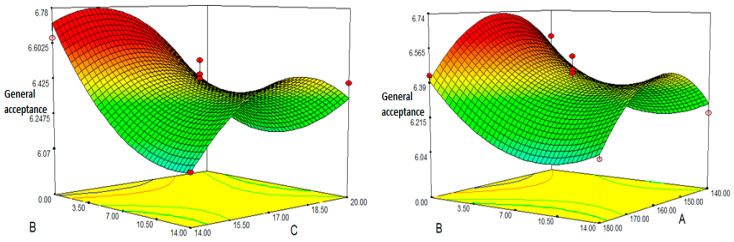
Response surface plot for the effect of fig molasses pomace content, moisture and temperature on general acceptance: (A) Temperature (°C); (B) Fig molasses by-product powder content (%); (C) Moisture content (wb%).

The linear and quadratic impacts of FMP content, die temperature, and moisture content on the final product were found to be significant (*p* < 0.05) in terms of appearance, colour, taste, and flavour (overall acceptability). Furthermore, after broad acceptance, it was found that there was a significant association (*p* < 0.05) from the interaction between the amount of FMP and the amount of moisture. The amount of FMP had a negative effect on overall acceptability (*p* < 0.05). Similar sensory ratings and decreases were observed in literature for carrot pulp and legume flour [35]. The increased amount of tomato peel has a negative impact on the textural features of extruded goods, such as hardness [71]. Furthermore, it was shown that the moisture content and the increase in die temperature had a detrimental impact on the sensory ratings. The increase in FMP, as seen above, may cause an increase in the hardness of the product due to an increase in the textural qualities of the product, particularly in the fibre and protein content; this impacts the crispness of the product. The square terms for barrel temperature, pulp content, and moisture content are significant, as shown by the F values and *p* values (*p* < 0.001) of parameters A^2^, B^2^, and C^2^. Acceptability rose quadratically as FMP content increased. FMP concentration also had a positive effect on the interaction term with moisture content; therefore an improvement in acceptability was found when the FMP ratio and moisture content increased. It was also discovered that changing the die temperature causes an increase or reduction in the acceptance level (Figure 7). The rise in temperature from 140 °C to 160 °C, as well as the rise in general acceptability, happened concurrently with the rise in the ER values of the snacks. Again, while the quantity of moisture initially had a good influence on general acceptability, greater increases in moisture diminished overall acceptability. A similar condition was discovered in the study that created extruded items from barley flour and carrot pulp [23]. The addition of FMP to corn semolina was consistent with the instrumentally assessed results and darkened the snack’s colour. The findings revealed that the amount of pulp has the greatest influence on sensory properties. Furthermore, our findings demonstrated that combining FMP and corn semolina can provide acceptable, unique, and healthy extruded snacks.

### 3.11. Optimal Extrusion Conditions

Design-Expert v7 was used to statistically optimize the independent variables. The maximum expansion rate and overall acceptability, as well as minimal firmness and bulk density, were found to be optimal parameters for the development of maize semolina-fig molasses pulp to make goods with a more pleasing texture. These traits were identified in the same way by prior researchers [12,72,73].

All extrinsic considerations were removed from this approach, and the remaining variables were left within range [37]. Eight formulations with estimated quality response values were developed based on the inputs supplied. Using the desirability function approach to cover our requirements, the generated solution manufactured with the optimum circumstances for manufacturing corn semolina-FMP extrudates has a desirability value of 0.724. Using data from the literature, it was determined that this value was within acceptable bounds [32]. The optimal snack manufacturing parameters were discovered to be 7% fig molasses pulp content, 155.44 °C die temperature, and 14.69% humidity. The predicted and actual values for the optimal manufacturing conditions are given in Table 6. A 95% confidence level t-test (IBM SPSS Statistics 22) was used to compare the estimated and actual values for snacks produced at optimum extrusion circumstances, and no significant difference was detected. To get the FMP snack extrudate with the requisite expansion, stiffness, bulk density, and general acceptability at the 7% pulp level, the results demonstrate that low feed moisture and moderate die temperature are required.

## 4. Conclusions

Increasing the efficiency of food processing, profitability and sustainability of food production requires the use of industrial by-products. In this study, an extruded expanded snack product with increased fibre content was developed by using process residues of fig molasses production which are not seen as having economic value and cause environmental problems if they are not disposed of under appropriate conditions. The components of fig molasses pulp have been set forth, above. This investigation is the first of its type and neither the fig fruit nor the manufacturing waste has ever been used in an extruded product. The total dietary fibre content of the products produced under ideal conditions was determined to be 2.04%. The study showed that the ideal parameters of 7% fig molasses pulp concentration, 155.44 °C die temperature, and 14.69% feed humidity may be used to produce extruded FMP-corn semolina snacks with satisfactory physical and sensory qualities. Consequently, it has come to light that a fig fruit by-product may be blended with corn semolina to make a beneficial product. Such snacks and research may aid in the future usage of FMP as a valuable resource for the manufacture of more sustainable and healthful products.

## Figures and Tables

**Table 1:** Some chemical properties of the raw materials mean scores used in the trials.

Component	FMP	Corn Semolina
Moisture (%)	5.19	11.49
Ash (%)	4.90	0.2–0.6
Protein (%)	13.50	5–9
Total dietary fiber (%)	53.01	<0.7
Total fat (%)	19.65	<1

FMP: Fig molasses by-product powder.

**Table 2:** Independent numerical variables and their levels.

Numerical Variables	Symbol	Coded Variable Levels		
		−1	0	1
Temperature (°C)	A	140	160	180
Fig molasses by-product powder level (%)	B	0	7	14
Moisture content (wb%)	C	14	17	20

A: Temperature (°C). B: Fig molasses by-product powder level (%). C: Moisture content (%wb).

**Table 3:** Experimental design layout.

	Coded Variables			Actual Variables		
Run	A	B	C	A (°C)	B (wb%)	C (%)
1	−1	0	1	140.00	7.00	20.00
2	0	−1	1	160.00	0.00	20.00
3	−1	1	0	140.00	14.00	17.00
4	1	1	0	180.00	0.00	17.00
5	0	1	−1	160.00	0.00	14.00
6	0	1	−1	160.00	14.00	14.00
7	−1	0	−1	140.00	7.00	14.00
8	0	1	1	160.00	14.00	20.00
9	−1	−1	0	140.00	0.00	17.00
10	0	0	0	160.00	7.00	17.00
11	0	0	0	160.00	7.00	17.00
12	1	0	−1	180.00	7.00	14.00
13	0	0	1	160.00	7.00	17.00
14	0	0	0	160.00	7.00	17.00
15	1	1	0	180.00	14.00	17.00
16	1	0	1	180.00	7.00	20.00
17	0	0	0	160.00	7.00	17.00

A: Temperature (°C). B: Fig molasses by-product powder level (%). C: Moisture content (%wb).

**Table 4:** Analysis of variance for response variables of extruded snacks.

	ER		WSI (%)		WAI		L		OA		Hardness (kg)			BD (g/cm^3^)
Source	Sum of Squares	*p*-Value	Sum of Squares	*p*-Value	Sum of Squares	*p*-Value	Sum of Squares	*p*-Value	Sum of Squares	*p*-Value	Sum of Squares	*p*-Value	Sum of Squares	*p*-Value
**Model**	6.676566	<0.0001 **	938.02	0.0007 **	3.41	0.0034 *	224.2575	<0.0001 **	0.97	0.0003 *	966.66	<0.0001 **	0.011	<0.0001 **
**A-Temperature**	0.91	<0.0001 **	478.80	0.0012 *	0.016	0.7484 ns	0.034716	0.8586 ns	0.039	0.0716 ns	85.87	0.0083 *	3.164 × 10^−3^	0.0001 **
**B-Fig molasses by-product powder level**	3.9214	<0.0001 **	9.53	0.5713 ns	2.30	0.0017 *	221.425	<0.0001 **	0.16	0.0027 ns	200.30	0.0004 *	1.065 × 10^−3^	0.0049 *
**C-Moisture content**	0.894453	<0.0001 **	449.70	0.0015 *	1.09	0.0177 *	2.797795	0.1268 ns	0.016	0.2219 ns	547.47	<0.0001 **	3.898 × 10^−3^	<0.0001 **
**AB**	0.12	0.0143 *												
**AC**	0.2	0.0030 *									365.5744	0.6599 ns	1.914 × 10^−3^	0.0007 **
**BC**	0.004761	0.5694 ns							0.12	0.0062 *	4303.885	0.1556 ns		
**A^2^**	0.13	0.0127 *							0.27	0.005 *	9571.715	0.0576 ns	5.336 × 10^−4^	0.0292 *
**B^2^**	0.31	0.0011 *							0.15	0.0033 *	6933.302	0.0818 ns		0.1297 ns
**C^2^**	0.21	0.0034 *							0.24	0.0007 *	133.02	0.0020 *	6.721 × 10^−4^	0.0171 *
**Residual**	0.098		367.19		1.93		13.66618		0.085		103.52		8.245 × 10^−4^	
**Lack of Fit**	0.072	0.1774 ns	298.09	0.2773 ns	0.91	0.8554 ns	11.16295	0.2660 ns	0.036	0.7062 ns	88.94	0.1481 ns	5.997 × 10^−4^	0.3003 ns
**Pure Error**	0.02639		69.10		0.96		2.503231		0.048		14.57		2.247 × 10^−4^	
**Cor Total**	6.77		1305.21		5.34		237.9237		1.06		1070.18		0.012	
**C.V. %**	2.87		12.79		9.05		1.445052		1.55		13.47		9.00	
**R-Squared**	0.985		0.7187		0.6389		0.942561		0.920		0.9033		0.9321	
**Adj R-Squared**	0.971		0.6538		0.5556		0.929305		0.857		0.8710		0.8914	

A: Temperature (°C). B: Fig molasses by-product powder level (%). C: Moisture content (%wb). C.V.: Coefficient variations. *: significant at *p* < 0.05. **: significant at *p* < 0.001. ns: not significant (*p* > 0.05).

**Table 5:** Pasting properties of FMP and semolina mixture.

Mixture Rate	PT (°C)	PV (cP)	FV (cP)	BV (cP)	SB (cP)
0	71.00 ± 0.52 ^a^	1193.99 ± 1.39 ^a^	5665.06 ± 125 ^a^	69.64 ± 5.2 ^a^	4540.71 ± 12 ^a^
7	73.88 ± 0.23 ^b^	480.26 ± 13 ^b^	1005.95 ± 16.91 ^b^	147.70 ± 3.7 ^b^	673.39 ± 7.5 ^b^
14	72.10 ± 0.89 ^a^	366.46 ± 14 ^c^	744.08 ± 27.21 ^c^	125.23 ± 2.81 ^c^	502.84 ± 15 _b_

Data are expressed as mean values with ± standard deviations of replicates. Different letters in the same column show significant differences (*p* < 0.05) according to the Tukey’s HSD comparison test. PT: pasting temperature; PV: peak viscosity; FV: final viscosity; BV: break down viscosity; SB: set back viscosity; cP: centipoise.

**Table 6:** Actual and predicted values of the optimum manufacturing conditions.

Responses	Actual Data	Predicted Data
ER	4.40 ± 0.17 ^a^	4.15 ^a^
BD (g/cm^3^)	0.071 ± 0.00 ^b^	0.081 ^b^
Overall acceptance	6.42 ± 0.44 ^c^	6.29 ^c^
Hardness (kg)	15.46 ± 0.62 ^d^	16.86 ^d^
WSI (%)	47.61 ± 0.75 ^ab^	45.53 ^ac^
WAI	4.48 ± 0.12 ^bc^	3.98 ^bd^
L*	71.02 ± 0.93 ^ab^	70.48 ^ab^

Data are expressed as mean values ± standard deviations. Different letters in the same line show significant differences (*p* < 0.05, *t*-test). N = 3. ER: Expansion ratio. BD: Bulk density. WSI: Water solubility index. WAI: Water absorption index. L*: Lightness.

## Data Availability

The data are available from the corresponding author.
